# Suburban vs urban: do the attendee's demographic profile influence the emergency department's mental health characteristics presentation?

**DOI:** 10.1192/bjo.2021.861

**Published:** 2021-06-18

**Authors:** Sudha Jain, Caoimhe Mcloughlin, John Cooney, Aoibheann McLoughlin, Ahad Abdalla, Siobhan MacHale

**Affiliations:** 1 Beaumont Hospital; 2 St James's Hospital; 3 Limerick University Hospital

## Abstract

**Aims:**

To compare the Emergency Department (ED) referrals to psychiatry in a suburban versus an urban setting over a one-month to evaluate mental health presentations characteristics across two locations.

**Method:**

This study was a retrospective cross-sectional study examining ED referrals to psychiatry in an inner-city and suburban centre over one month; - one based in an inner-city setting, the other based in a suburban area outside the city. The anonymised data were collected from both hospital's electronic patient records and analysed. The authors collected data on gender, age, employment, housing, the clinical problem at presentation, time of assessment and admissions. Descriptive data and hypothesis testing were performed where appropriate using Statistical Package for Social Sciences SPSS® version 26.

**Result:**

The total number referred was 213: inner-city n = 109 and suburban n = 104. The inner-city saw a younger population; 47/109 (43%) were aged between 20 and 29 years, compared with 28/104 (27%) of suburban presenters (P-value 0.0134). A higher number of presenters were aged over 60 years in the suburban centre n = 13/104 (12.5%) versus the inner-city centre 3/109 (2.8%) (P-value 0.0084). In the inner-city, the proportion of homeless presenters was significantly higher at 30/109 (28%) versus 5/104 (4.8%) in the suburban setting (P < 0.0001). Presentations related to substances were highest, a total of 73 (34.3%) across both centres, with no significant difference in clinical presentations across the two centres. The majority were seen in the on-call period, 74/109 (67.9%) in the inner-city centre and 66/104 (63.5%) in the suburban centre. The psychiatric admission rate was significantly different between the two centres, with 33/109 (30.3%) patients admitted to the inner-city centre and 13/104 (12.5%) patients admitted to the suburban centre (P-value 0.002).

**Conclusion:**

A large proportion of ED referrals to psychiatry constitute patients with unmet social and addiction needs. The variance in capabilities of liaison psychiatry (LP) and ED services means the local population's needs may not always be adequately catered for within a typical LP setting, which in the Irish context is predominantly driven by medical and nursing staff. This study highlights many patients attend the ED who may be better assessed directly by the community as per our National Emergency Program policies. This prompts consideration of expanding both ED and community services to comprise a more integrated, multidisciplinary-resourced, 24/7 care model.

